# Effect of screw access hole preparation on fracture load of implant-supported zirconia-based crowns: an *in vitro* study

**DOI:** 10.15171/joddd.2016.029

**Published:** 2016-08-17

**Authors:** Hadi Mokhtarpour, Reza Eftekhar Ashtiani, Minoo Mahshid, Farhad Tabatabaian, Marzieh Alikhasi

**Affiliations:** ^1^Assistant Professor, Department of Prosthodontics, Faculty of Dentistry, Sari University of Medical Sciences, Sari, Iran; ^2^Assistant Professor, Department of Dental Technology, School of Dentistry, Shahid Beheshti University of Medical Sciences, Tehran, Iran; ^3^Professor, Department of Prosthodontics, School of Dentistry, Shahid Beheshti University of Medical Sciences, Tehran, Iran; ^4^Assistant Professor, Department of Prosthodontics, School of Dentistry, Shahid Beheshti University of Medical Sciences, Tehran, Iran; ^5^Associate Professor, Department of Prosthodontics, School of Dentistry, Tehran University of Medical Sciences, Tehran, Iran

**Keywords:** Crown, dental implant, implant-supported dental prosthesis, zirconia

## Abstract

***Background.*** Fracture load of implant-supported restorations is an important factor in clinical success. This study evaluated the effect of two techniques for screw access hole preparation on the fracture load of cement-screw-retained implant-supported zirconia-based crowns.

***Methods.*** Thirty similar cement-screw-retained implant-supported zirconia-based maxillary central incisor crowns were evaluated in three groups of 10. Group NH: with no screw access holes for the control; Group HBS: with screw access holes prepared with a machine before zirconia sintering; Group HAS: with screw access holes prepared manually after zirconia sintering. In group HBS, the access holes were virtually designed and prepared by a computer-assisted design/computer-assisted manufacturing system. In group HAS, the access holes were manually prepared after zirconia sintering using a diamond bur. The dimensions of the screw access holes were equal in both groups. The crowns were cemented onto same-size abutments and were then subjected to thermocycling. The fracture load values of the crowns were measured using a universal testing machine. Data were analyzed with ANOVA and Tukey test (P < 0.05).

***Results.*** The mean fracture load value for the group NH was 888.37 ± 228.92 N, which was the highest among the groups, with a significant difference (P < 0.0001). The fracture load values were 610.48 ± 125.02 N and 496.74 ± 104.10 Nin the HBS and HAS groups, respectively, with no significant differences (P = 0.44).

***Conclusion.*** Both techniques used for preparation of screw access holes in implant-supported zirconia-based crowns decreased the fracture load.

## Introduction

Implant-supported restorations are considered a choice for replacing missing teeth. Selection of the type of attachment among different implant prostheses and implants plays a pivotal role in the clinical service and long-term success of an implant-supported restoration. Retention of implant-supported crowns may be provided by the use of a screw or cement. Screw-retained and cement-retained restorations have benefits as well as shortcomings. For instance, crown retrieval is easier in screw-retained restorations, but the crown may not be passively placed over the abutment, and the screw may become loose or broken.^1,2^ Cement-retained restorations have advantages such as a passive fit and affordability. However, they have some limitations, including early loosening, difficult or impossible retrieval, and impossible cleaning of excess cement in deeply placed implants.^2,3^

Currently, due to the improved design and a better understanding of the mechanics of screw, screw loosening or fractures are rare in dental implants.^4^ However, because of the increased patient demand for implant treatment, the prevalence of screw loosening and its associated biological and technical complications is on the rise.^5^According to a systematic review conducted by Jung et al,^6^ problems of the implant/abutment joint, such as screw loosening and fracture, are among the most common complications compromising the survival of implant-supported, screw-retained fixed partial dentures.^6^

Several methods have been proposed for the retrieval of cement-retained implant-supported crowns. Chee et al^7^ introduced a technique using a screw locked in the implant restoration to retrieve the cemented restoration. Okamoto et al^8^ suggested preparing cylindrical holes in the lingual surface for this purpose. McGlumphy et al^9^ suggested providing retention by both cement and screw, to facilitate accessing the screw hole of restorations and to enable cleaning of the subgingival cement. This technique was referred to as the “combination implant crown”.^10^This method has been recommended for use in implant-supported metal ceramic restorations; it also allows for permanent cementation of crowns instead of using a temporary cement.^6,9,10^Baige et al^11^ reported a technique to retrieve implant-supported cemented restorations that benefits from the advantages of both cement-retained and screw-retained fixations. A case report by Rojas-Vizcaya^12^ described restoration of a maxillary lateral incisor via a cement-screw-retained implant-supported zirconia-based crown. In this method, a screw-retained metallic substructure was designed and a cement-retained crown was fabricated over it. It seems that cement-screw-retained implant-supported restorations have been well developed in prosthodontics.

On the other hand, implant-supported zirconia-based crowns, fabricated by computer-assisted design/computer-assisted manufacturing (CAD/CAM) systems, have acceptable esthetics, optimal biocompatibility, high fracture strength, resistance against crack propagation and high adaptation, which explains their popularity. Worni et al^13^ showed that treatment with implant-supported zirconia-based fixed prostheses yielded favorable outcomes and that screw retention was feasible at the implant level. However, these prostheses have to be screwed and suffer from the same limitations as metal-ceramic cemented crowns.^3^ Use of cement-screw-retained implant-supported zirconia-based restorations can be a solution to this problem. In this approach, a zirconia-base restoration with a screw access hole is fabricated, cemented on a titanium abutment in the lab, and finally screwed onto a dental implant in the oral cavity.

Considering the structure of zirconia-based crowns and the mechanism of crack propagation and its control,^14^the effect of screw hole preparation on the crown fracture load has yet to be clearly understood. Some previous studies on the fracture load of cement-retained and screw-retained implant-supported metal-ceramic restorations with and without screw access holes have shown that crowns with screw access holes had lower fracture load.^15,16^ In a clinical setting, the mechanical resistance of implant-supported restorations plays a pivotal role in the clinical service and long-term success of dental implants.^17^ The fracture load is defined as the maximum force that a material can tolerate before fracture. Several factors influence the fracture load of zirconia ceramics, including loading conditions, type of cement, and restoration design, the density of pre-sintered zirconia blocks, zirconia sinterability, mechanically created cracks, residual compressive stresses during preparation, yttria contents of zirconia, external stresses due to sandblasting, wear, cyclic loading and thermal or chemical aging .^18^

Despite some previous studies related to the fracture load of zirconia ceramics,^19-24^ the effect of screw access hole preparation on the fracture load of zirconia-based crowns has yet to be fully evaluated. Hence, the aim of this study was to assess the effect of two techniques for screw access hole preparation on the fracture load of implant-supported zirconia-based crowns. The null hypothesis was that screw access hole preparation would not affect the fracture load of implant-supported zirconia-based crowns.

## Methods

This was an in vitro study which did not involve the use of any animals or human data or tissues, and thus an ethics approval was not required.

### Specimen preparation

**Figure 1.**
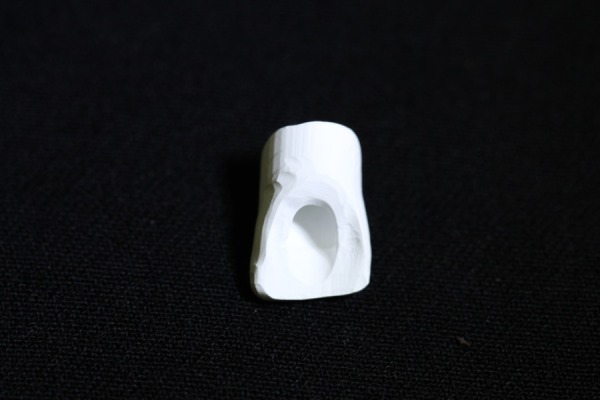
A milled sample of zirconia core with screw access hole before sintering.

**Figure 2.**
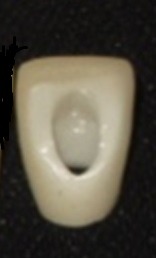
The metal mold used for veneering process.

**Figure 3.**
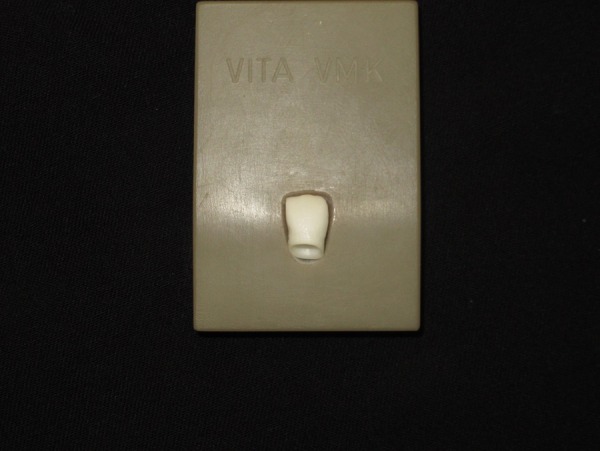
The palatal view of a prepared zirconia-based crown before cementation.

Three implant analogs of the Noble Replace straight internal tri-channel dental implants (Nobel Biocare, Goteborg, Sweden), measuring 4.3mm in diameter and 15mm in height, were used in this experimental study. Three 15° esthetic abutments (Nobel Biocare, Goteborg, Sweden), measuring 0.5 in mm collar height and 7.5 mm in height, were selected. The abutments were fixed in the implant analogs by applying a torque of 35 N.cm based on the manufacturer’s instructions. A two-piece custom metal block was fabricated and the implant analogs were then embedded in the metal block at a 35° angle relative to the vertical axis. Taking into account the 15° angulation of the abutment relative to the longitudinal axis of implant analog, the final angulation of abutment relative to the vertical axis was 50°.^22^ Since all the abutments had equal dimensions, a wax pattern of an anterior crown without a screw access hole was made on the abutment for laser scanning using the Sirona inEos machine (Sirona, Bensheim, Germany).The wax pattern was scanned and the scanned image was transferred to the Sirona inEos CAD/CAM machine (Sirona, Bensheim, Germany).Then a screw access hole with predetermined dimensions (maximum mesiodistal and buccopalatal widths of 3 mm and 4.5 mm, respectively) was created on the same wax pattern and scanning process was performed again. The scanned images were used to fabricate 30 zirconia cores with the thickness of 0.6 mm from zirconia blanks (Whitepeaks, Copran Zr-i, Wesel, Germany) (20 cores without screw access hole and 10 cores with screw access hole) (Figure 1). The zirconia cores (n=30) were sintered at 1480°C for an eight-hour process in a sintering furnace (Sintramat, Ivoclar Vivadent, Germany ) according to the instructions recommended by the manufacturer. For ten zirconia cores without screw access hole, the holes were manually prepared on the palatal surface using a coarse round-end taper diamond bur (ZR Diamond, Komet Besigheim, Germany). To match the dimensions of machinery and manually created screw access holes, a silicone index adapted to the palatal surface was used. Also a digital caliper evaluated the mesiodistal and buccolingual widths of the holes. In case of differences more than 0.1 mm, the zirconia core was excluded from the study. The zirconia cores were divided into three groups: one group without screw access holes for the control and two experimental groups with screw access holes. The zirconia cores were evaluated to ensure uniform thickness of 0.6±0.02 mm at different areas using a digital micrometer. Furthermore, the zirconia cores were tested by an explorer and a silicone material for their fitness. Also they were evaluated radiographically for marginal adaptation. Finally all the zirconia cores were veneered using a central incisor multi-piece metal index with specific dimensions (11mm in height, 8mm in width) (Figure 2)with a silica-based ceramic veneer (Vintage Shofu-ZR, Kyoto, Japan). The veneer thicknesses were 2 mm for incisal, 1.5mm for mid-labial, and 0.5mm for cervical and palatal surfaces. The zirconia-based crown specimens were fabricated as such (Figure 3) and divided into three groups as follows:

**Group NH:** This group included 10 zirconia-based crowns with no screw access holes.

**Group HBS:** This group included 10 zirconia-based crowns with screw access holes which were prepared before zirconia core sintering. The holes were designed by the CAD/CAM system according to the scanned image, with predetermined dimensions. The zirconia cores were sintered and veneered.

**Group HAS:** This group included 10 zirconia-based crowns with screw access holes which were prepared after zirconia core sintering. The holes were manually prepared in the palatal surface of zirconia cores using the mentioned bur, and matched dimensionally to group HBS.

The abutments were cleaned with pumice powder and water. Cotton pellets were placed over the abutment screws. For specimens in group NH, the abutment screw holes were filled with composite resin (Z250 Restorative, 3M ESPE, St. Paul, Minn). Next, the composite resin was light-polymerized prior to cementation. All the thirty crowns were cemented over the abutments using resin cement (Panavia 21,Kuraray, Tokyo, Japan) according to manufacturer’s instructions and pressed with finger pressure for 2 minutes.^23^The excess cement was removed. For specimens in groups HBS and HAS, after cementation, the screw access holes were completely filled with the same composite resin and the composite resin was light-polymerized. All the specimens were subjected to 500 thermal cycles in a thermocycler between 5 and 55°C, with a dwell time of 60 seconds for each specimen and 12 seconds of intermediate pause.

### Testing

**Figure 4.**
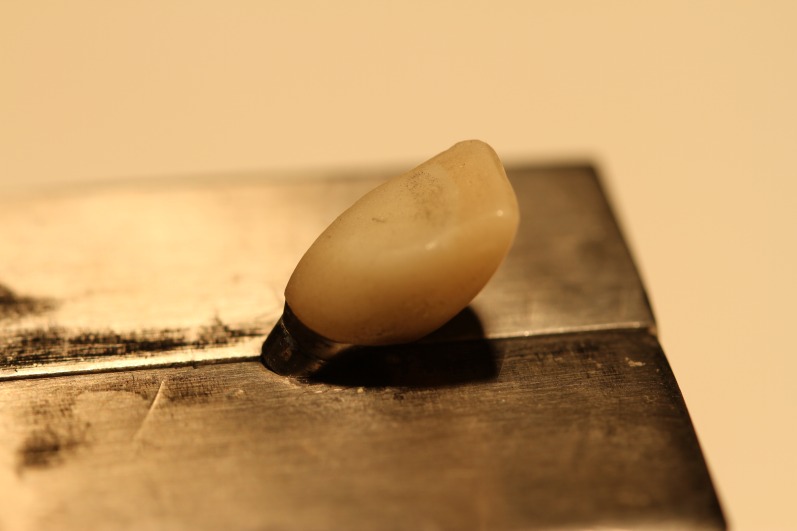
A prepared sample of the NH group for testing.

To determine the fracture load, the specimens were subjected to compressive load testing in a universal testing machine (Z0-50, Zwick Roell, Ennepetal, Germany). Compressive loads were applied vertically and at a 50° angulation relative to the abutments at a crosshead speed of 2 mm/min (Figure 4).^22,24^ The load was applied 3 mm below the incisal edge^23^ out of the hole area. The load applicator of the universal testing machine was similar to the incisal edge of a mandibular incisor (dimension of 5.5×1 mm), to simulate clinical settings.^25^ The magnitude of the load, which was applied by the universal testing machine, increased until the zirconia core fractured. The fracture load was recorded. In case of ceramic veneer fracture without core fracture, the specimen was eliminated from the study. After fracture occurred, the specimens were heated to decrease the retention of cement on the abutment, and the crown was removed. After removal of the crown, the cement remaining on the abutment was thoroughly removed by an explorer. The abutment surface was cleaned with an acetone solution and water. Then the abutment was dried, tightened using a torque of 35 N.cm, and prepared to cement the next crown. This process was repeated for all the specimens. The fracture load was measured for each specimen in Newton (N). Data were analyzed with ANOVA and Tukey test using SPSS 20 (SPSS Inc., Chicago, Ill). P < 0.05 was considered statistically significant.

## Results

**Figure 5.**
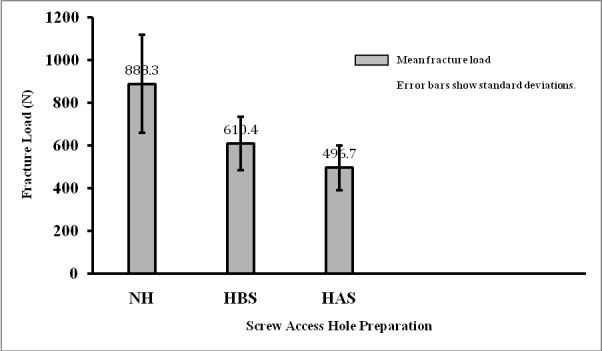
The graph demonstrates means and stan-dard deviations of fracture load values for the three groups.

The means and standard deviations for the fracture load values of the specimens in the three groups were 888.3 ± 228.9, 610.4 ± 125, and 496.7 ± 104.1 Nin the NH, HBS and HAS groups, respectively (Figure 5). After ensuring a normal distribution of data using the non-parametric Kolmogorov-Smirnov test, one-way ANOVA was employed to compare the fracture load values of the three groups. The results revealed a statistically significant difference in fracture load values between the three groups (P < 0.0001). The results of Tukey HSD test revealed significant differences in the fracture load values between the NH and HBS groups (P < 0.001) and also between the NH and HAS groups (P < 0.0001). However, the difference between groups HBS and HAS was not significant (P = 0.44).

## Discussion

Based on the results of the current study, preparation of screw access holes in cement-screw-retained implant-supported zirconia-based crowns before and after sintering (machined and manually) resulted in a significant reduction in the fracture load values. Moreover, considering the technique of the preparation, the manual and machine techniques gave rise to no differences in this respect. Thus, the null hypothesis of the study was refuted.

Cement systems, bonding procedures, occlusion and metal copings all play a role in the success of fixed prostheses and all these factors must be taken into account in order to be able to make a comparison among novel ceramic and metal substructures.^26^Therefore modifications on the substructures may affect the fracture of restorations. One of these modifications is to prepare screw access holes. Most relevant previous studies have evaluated the effect of access hole preparation on the fracture load of posterior metal-ceramic restorations and to the best of authors’ knowledge, no similar study has been conducted on cement-screw-retained implant-supported zirconia-base crowns.

Several studies have demonstrated that preparation of screw access holes in screw-retained metal-ceramic restorations decreased the fracture load of porcelain.^16,27^ Some researchers believe that the preparation of screw access holes in screw-retained restorations can undermine the ceramic around the hole and also the ceramic at the cusp tip, leading to ceramic fracture; however, this problem does not exist in cement-retained restorations.^15,28^Evidence has shown that cement-retained restorations with intact occlusal surfaces have higher porcelain fracture load than screw-retained restorations.^15,29,30^ Similar results were obtained in the current study since the preparation of screw access holes before and after sintering significantly decreased the fracture load of zirconia crowns. Wood et al^31^evaluated the effect of access cavity preparation for root canal therapy on the fracture load of zirconia and alumina restorations. All the specimens showed porcelain chipping around the access cavity and the decrease in the fracture load of the zirconia restorations was greater than that of the alumina restorations.^31^ However, it should be noted that in the study by Wood et al,^31^the access cavity prepared for root canal therapy was much larger than the screw access hole prepared in implant-supported restorations. This difference may have been responsible for the inconsistent comparisons. Screw access hole preparation in screw-retained restorations disrupts the structural integrity of the zirconia ceramic and results in subsequent displacement of the center of ceramic mass towards ceramic gaps during the sintering process. Such a displacement changes the behavior of ceramic in these restorations compared with cement-retained restorations.^30^ Nonetheless, it should be noted that due to the variability in the use of different methods, implant systems, loading conditions, cement materials, restoration design, type of abutment and type of alloy, a direct comparison of the results across different studies may not be feasible.

Fully sintered zirconia is very tough, making its preparation extremely difficult. Moreover, the preparation of zirconia ceramic has adverse effects on its mechanical properties.^19^The strength of zirconia directly relates to the size, number, location and distribution of defects, cracks and superficial gaps/porosities.^32^ However, mass cracks possess a covering protection and are supported by the surrounding material. Superficial cracks directly serve as stress accumulation areas and intensify the applied stresses, depending on their extent.^32^This is due to the size of superficial cracks in all-ceramic restorations. Sharp, deep defects result in an increased concentration of stress at the tip of the cracks and likely serve as sites of crack initiation.^33^ Although the superficial defects created in the process of CAD/CAM preparation may have insignificant effects on the strength of specimens, they may have a significant impact on the fatigue behavior of zirconia restorations.^34^ Small defects propagate due to cyclic loading of mastication and eventually cause sudden fractures in ceramic.^34^ Consequently, despite their small size, superficial defects can serve as stress concentration sites and can lead to restoration fracture.^34^

Roebben et al^35^ reported that many factors can affect the mechanical properties of zirconia ceramics such as hardness and strength. These factors include the density of pre-sintered zirconia blocks (due to its correlation with critical crack size), sinterability of the powder (due to its correlation with the size of primary particles), mechanically created cracks, residual compressive stresses during preparation of specimens and high yttrium content (due to its significance in transformation of tetragonal to monoclinic phase following stress application).^36^Consequently, the screw access hole preparation may mechanically create cracks. This may be the reason for a decrease in fracture load following screw access hole preparation in the present study.

In the current study, the thickness of the zirconia core was adjusted at 0.6mm according to a study by Potiket et al.^36^ Denry et al^37^suggestedthat the minimum thickness of zirconia core should be 0.5 mm to avoid fractures; accordingly, a durable zirconia core thickness was employed in the present investigation. Haraldson et al^38^reported a mean load of 206 N applied to the anterior region of dental arch during masticatory function. Maximum bite forces in the anterior incisor region correspond to approximately 150 Nin adults.^39^ Gay et al^40^ estimated human incisal bite forces ranging from a minimum of 25 N to a maximum of 400 N. Although the results of the present study cannot be directly generalized to a clinical setting, all the study groups showed higher fracture loads than the anterior biting force. Based on the results of this study and also the range of occlusal loads applied to the anterior teeth, the fracture load values of the study groups were within an optimal range of functional loads. Similarly, Worni et al^13^ demonstrated that screw-retained implant-supported zirconia crowns had optimal survival rates in the oral environments.

The current study had some limitations; the crowns studied were only subjected to one cycle of compressive load until fracture; whereas, in a clinical setting, the crowns may undergo fracture following fatigue processes and the formation of small cracks.^41^ Obviously, application of a single, compressive load cannot simulate all the clinical loads that a restoration is exposed to.^42^ In fact, one may not be able to exactly relate a fracture load test, performed with the application of single compressive load until fracture in a dry environment, to the dynamics of numerous fractures that occur in the wet oral environment due to cyclic loading.^43^ Therefore, generalization of the results would have been possible if the specimens had been tested under fatigue loading. Moreover, quick load application to a single point is different from normal mastication in the oral environment because the clinical pattern of loading decreases the loads applied to the restorations.^44^ Furthermore, a clinical pattern of load application can cause fatigue fractures in small amounts compared to in vitro loading conditions. Therefore, further studies are necessary under the conditions noted above. If the results of the current study can be supported by further successful clinical researches, screw access hole preparation will be deemed a useful method to fabricate cement-screw-retained implant-supported zirconia-based restorations.

Based on the results of the present study in relation to a decreased fracture load in cement-screw-retained implant-supported zirconia-based crowns, reinforcement of the zirconia core is recommended in these cases. Also screw access hole preparation techniques need innovations and more investigations.

## Conclusions

Within the limitation of the study it was concluded that:

1. The preparation of screw access hole in cement-screw-retained implant-supported zirconia-based crowns before sintering (machined technique) and after sintering (manual technique) decreased the fracture load.
2. In this respect, there was no significant difference between the two techniques of access hole preparation (manual and machined).

## Acknowledgements

The authors do not have any individual or organization to acknowledge with regards to this paper.

## Authors’ contributions

HM, REA, and MM contributed to the concept and design of the work. The acquisition, analysis, and interpretation of data were accomplished by MM and FT. HM, MM, FT, and MA drafted and revised it critically for intellectual content. All the authors read and approved the final manuscript.

## Funding

This project was supported and funded by the authors.

## Competing interests

The authors declare no competing interests with regards to the authorship and/or publication of this article.

## Ethics approval

Not applicable.
